# Memorial Service for Nurses Who Have Fallen in the War

**Published:** 1918-04-13

**Authors:** 


### ST. PAUL'S CATHEDRAL.

### MEMORIAL SERVICE FOR NURSES WHO HAVE FALLEN IN THE WAR.

St. Paul's Cathedral, which the preacher on April 10, the Venerable Archdeacon Holmes, denominated the Parish Church of the British Empire, is indeed a centre of splendid organisation, worship, and convinced Christianity, the influence of which has grown exceedingly since the war began. It has been our privilege to attend many services there since 1914, and on each occasion the impression carried away by the congregation has been one of thankfulness for and appreciation of the inspiration and oneness of the services and the music, with a feeling of gratitude for the effect of its atmosphere upon the people assembled within its walls not only on great national occasions but invariably. The Memorial Service for Nurses who have fallen in the war was no exception to the universal experience just recorded, and it had new features and marked excellence in more than one direction. Never before has the Parish Church of the British Empire been filled to overflowing with nurses grouped together in each case under their leader, wearing the uniforms and badges of the various Services to which they are attached, so that the massed effect was striking and remarkable.

Archdeacon Holmes must have had this united effect driven home to him when he gazed from the pulpit on all these nurses grouped in brigades and extending practically from the chancel steps right up to the western door. The application of his text, "Not one of them is forgotten before God" (Luke xii. 6) made this clear from the first. He drove home and insisted with great energy the truth that not one of the nurses fallen in the war is forgotten. Not one of the five members of Queen Alexandra's Imperial Military Nursing Service. Not one of the sixty-nine nurses of Queen Alexandra's Imperial Military Nursing Service Reserve. Not one of the four nurses of Queen Alexandra's Royal Naval Nursing Service and its Reserve. Not one of the three Colonial Nursing Association nurses. Not one of the twenty-nine nurses of the Territorial Force Nursing Service. Not one of the eleven British Red Cross and St. John nurses. Not one of the twelve nurses of the Scottish Women's Hospitals. Not one of the eight nurses of the Australian Army Nursing Service. Not one of the seven Canadian Army Medical Service Nurses. Not one of the two nurses of the Indian Military Nursing Service. Not one of the fourteen New Zealand Nursing Service nurses. Not one of the three South African Army Nursing Service nurses. Not one of the four members of the Serbian Relief Fund. Nor are Nurse Clementina Addison, who died in France, and Nurse Edith Cavell, executed in Belgium, forgotten. Not one of the 183 members of Voluntary Aid Detachments is forgotten. All are typical of the splendid spirit and work of British women everywhere since the war began in 1914, and of the pioneer nurses who accompanied the First Seven Divisions, sharing with our warriors the strain and dangers of our first Expeditionary Force in France whose services and deeds will be memorable so long as the British race endures.

Addressing the nurses present Archdeacon Holmes reminded them that there was not one of them or one of their sisters whom they represented, from the youngest probationer to the greatest of matrons, including sisters and charge nurses of all types, who is not held in remembrance, or the glory of whose work can be taken away from her or her sisters, so long as they are faithful to their exemplar, the Madonna, a fact they should ever treasure in their memories. Within St. Paul's Cathedral, in the crypt, was one memorial to a woman, the only woman ever commemorated in St. Paul's—Florence Nightingale. He was not there to praise nurses. It would be an insult to them to do so. But he did wish to remind them of the Empire's gratitude which their work had yielded, and that their brave, fearless, noble sisters, whose death they were commemorating at that service, had a message for every nurse present and throughout the Empire, a message which each should bear to her sisters outside. That message was: "Fill up the ranks. Carry on." Nurses were now everywhere regarded with reverence. He hoped they would always do their utmost to sustain that feeling towards their profession and themselves. Every nurse should study to make herself felt as a woman. Queen Victoria throughout her whole reign possessed this capacity and exercised it, to the great benefit of her people. It was the secret of the love her people bore for her, and of the reverence which she excited from her subjects. Matrons, sisters, and all types of nurses should show reverence habitually, and take Queen Victoria as their example of conduct and aspiration, that reverence might be theirs.

When his voice ceased to be heard they would hear the roll of the drums and the music of the "Dead March" in "Saul." Those who heard them for the first time in the Cathedral must be impressed and thrilled, and all, however familiar they might be with that roll, would welcome it and bear it away as a treasured memory recalling the present occasion and its purpose throughout their lives. "Remember this roll-call. Let it inspire you to reverence, keeping constantly before you the message I have brought you to-day—'Fill up the ranks. Carry on.'"

The preacher compared the German woman in Red Cross garb who took a cup of water to a dying British soldier, and as it was near his lips emptied it out on the ground and left him racked with thirst, to Jezebel, much to Jezebel's advantage. This thrilled the vast audience, for everybody present felt the act justified the speaker, though he was standing in the pulpit of the Parish Church of the British Empire.

The band of the Coldstream Guards, under the

**MEMORIAL SERVICE FOR NURSES**—(*continued*).

direction of Major J. Mackenzie Rogan, M.V.O., Mus. Doc., before the service played Arthur Somervell's elegy "Killed in Action," Gounod's "There is a green hill far away," and Sir Arthur Sullivan's overture, "In Memoriam." The last was splendidly rendered, most impressive, and appropriate. The "Dead March" in "Saul" (Handel) was never heard to greater advantage or better played. The roll of the drums and the splendid conductorship of Major Mackenzie Rogan will make it linger in the memory of all present. Then came the "Last Post," sounded by the drummers of the 5th (Res.) Battalion Coldstream Guards. They were stationed just within the west door of the Cathedral, and following as it did the "Dead March" and filling the Cathedral, its effect was stirring to the quick, and the last bars of farewell were so realistic as to sound as if spoken to the listener.

The Empire owes generous thanks to the authorities of St. Paul's Cathedral, and everyone present was warm in gratitude to the Rev. A. Lombardini for his splendid organisation and the perfection of the arrangements for marshalling the audience and securing everybody the seat previously allotted to them.

